# Supplementary material for: Multisensory coding of angular head velocity in the retrosplenial cortex
Source: Neuron. 2022 Feb 2;110(3):532–543.e9. doi: 10.1016/j.neuron.2021.10.031 (PMC8823706; doi:10.1016/j.neuron.2021.10.031)
Supplement: Document S1. Tables S1–S2 and Figures S1–S8 [file mmc1.pdf]

Neuron, Volume *110*

Supplemental information

Multisensory coding of angular head velocity in the retrosplenial cortex

Sepiedeh Keshavarzi, Edward F. Bracey, Richard A. Faville, Dario Campagner, Adam L. Tyson, Stephen C. Lenzi, Tiago Branco, and Troy W. Margrie

Table S1. Parameters used for automated spike sorting with KiloSort. Related to STAR Methods.

ops.Nfilt	640*
ops.whitening	full
ops.nSkipCov	1
ops.whiteningRange	32
ops.criterionNoiseChannels	0.1
ops.Nrank	3
ops.nfullpasses	6
ops.maxFR	20000
ops.fshigh	300
ops.ntbuff	64
ops.scaleproc	200
ops.Th	[4 10 10]
ops.lam	[5 20 20]
ops.nannealpasses	4
ops.momentum	1./[20 400]
ops.shuffle_clusters	1
ops.mergeT	0.1
ops.splitT	0.1
ops.initialize	fromData
ops.spkTh	-4
ops.loc_range	[3 1]
ops.long_range	[30 6]
ops.maskMaxChannels	5
ops.crit	0.65
ops.nFiltMax	10000

* Neuronexus Poly2 = 64

Table S2. Parameters used for automated spike sorting with KiloSort2. Related to STAR Methods.

ops.fshigh	300
ops.minfr_goodchannels	0
ops.Th	[10 4]
ops.lam	10
ops.AUCsplit	0.85*
ops.minFR	1/50
ops.momentum	[20 400]
ops.sigmaMask	30
ops.ThPre	8
ops.spkTh	-6†
ops.nfilt_factor	4
ops.ntbuff	64
ops.whiteningRange	32
ops.nSkipCov	25
ops.scaleproc	200
ops.nPCs	3

* Neuronexus Poly2 = 0.6

† Neuronexus Poly2 = -3

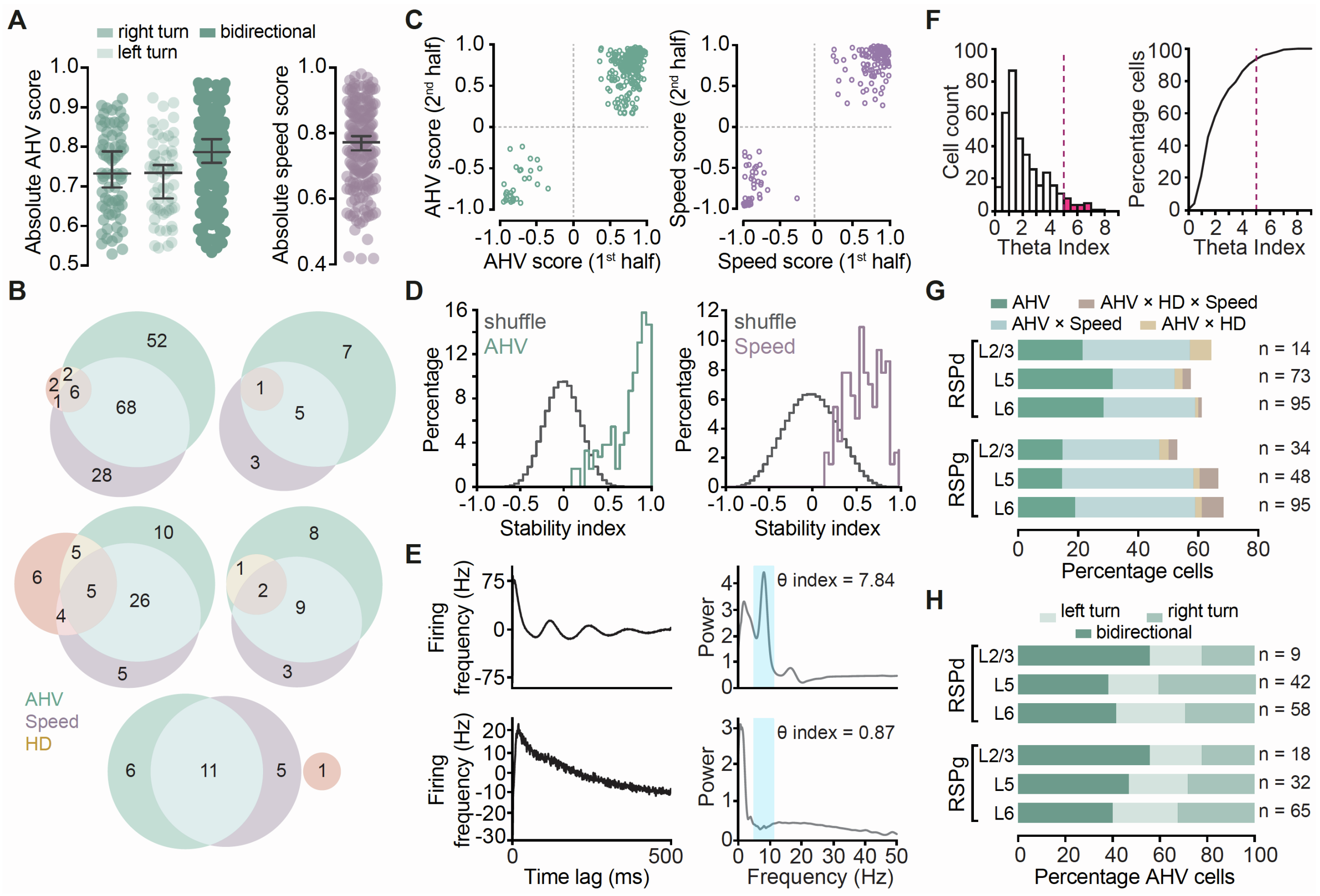

Figure S1. Tuning Properties of RSP neurons recorded during free exploration. Related to Figure 1.

A, Magnitude of AHV (left) and speed (right) scores, defined as absolute Pearson's r of correlations between a cell's firing rate and AHV or linear locomotion speed. Each circle represents a tuned cell. Black lines and error bars are median and 95% CI. Neurons with bidirectional AHV tuning (dark green) are represented by two circles for the two turning directions. Neurons with unidirectional AHV tuning are shown in transparent green shades (right turn or left turn) **B**, Venn diagrams showing cells tuned to head direction (HD), AHV, and linear locomotion speed in each mouse. **C**, AHV (left) and speed (right) scores from the first half of the recording session plotted against that of the second half for each tuned cell (AHV Spearman rho = 0.6, p <1e-15; Speed Spearman rho = 0.72, p <1e-15). **D**, Distribution of stability indices for AHV (left, green) and speed (right, purple) tuned cells versus shuffled distributions (grey). **E**, Autocorrelogram (left, bin size = 1 ms) and power spectrum (right) of a theta modulated cell (top) and a cell with no theta rhythmic firing (bottom). Blue window defines 5 - 11 Hz theta range. **F**, Left: frequency distribution of theta indices for all RSP neurons recorded in the open field. Pink bars show the number of theta modulated cells. Right: cumulative distribution of theta indices in the population. Approximately 94% of RSP cells did not show theta rhythmic firing. Dashed lines delineate the cut-off for a significant theta index. **G**, Percentage of AHV tuned neurons across RSP regions and layers. n, number of recorded neurons in each layer. **H**, Percentage of AHV cell types across RSP regions and layers. n , number of AHV tuned neurons in each layer.

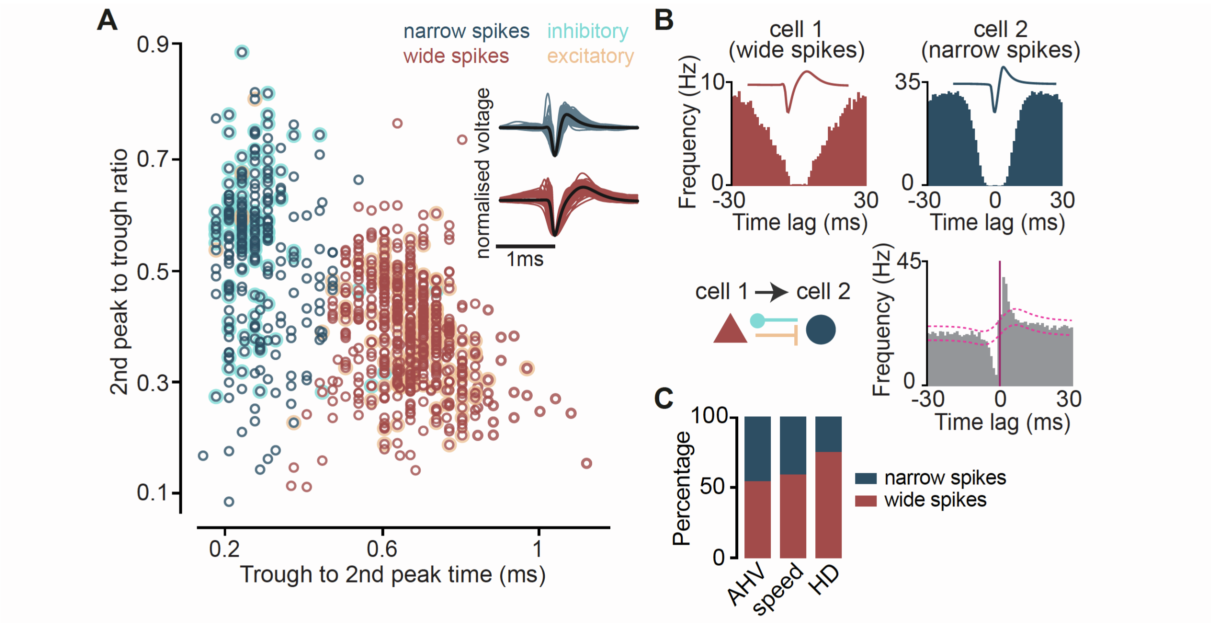

Figure S2. Both excitatory and inhibitory neurons in the RSP represent AHV. Related to Figure 1.

A, K-means clustering of isolated RSP units based on spike width and peak/trough ratio. Single units were separated into two clusters with wide (putative excitatory, n = 460, 68%, red) and narrow (putative inhibitory, n = 216, 32%, dark blue) spikes. Cyan and orange circles mark neurons identified as inhibitory and excitatory based on cross-correlogram (CCG) analysis. **B**, Top: average spike waveform and auto-correlogram of two example single units with wide and narrow spikes. Bottom: schematic of reciprocal monosynaptic connections (left) based on the CCG for the same pair (right). Dashed lines indicate 99.98% confidence interval. **C**, Percentage of putative excitatory (wide spikes) and inhibitory (narrow spike) neurons among AHV (n = 224), speed (n = 182), and HD (n = 36) tuned cells.

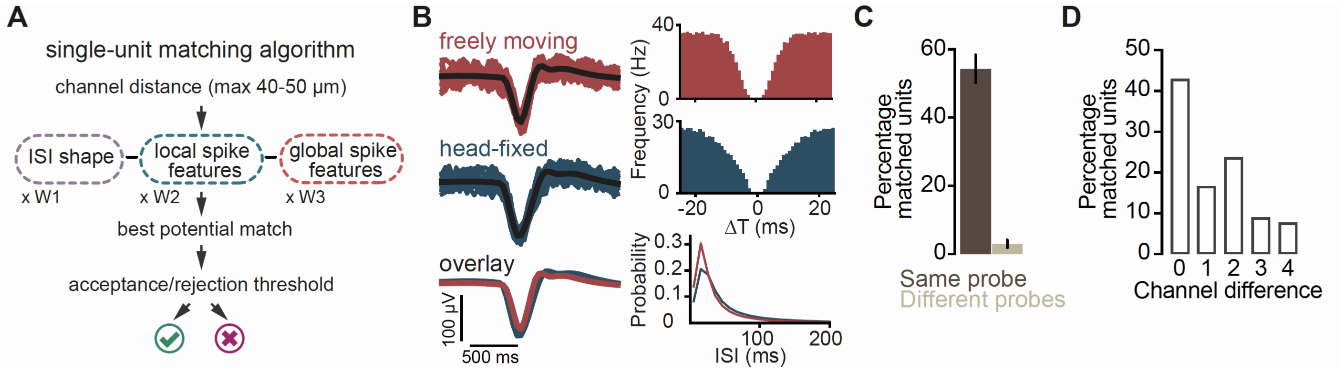

Figure S3. Tracking single units between freely moving and head-fixed recordings. Related to Figure 2.

A, Schematic of the single-unit matching algorithm. Probe geometry and multiple similarity metrics for spike features and inter-spike interval (ISI) distributions were used to find best potential matches. **B**, Example of a single unit tracked between head-fixed (blue) and freely moving (red) sessions. Top- and centre-left: red and blue traces are individual spikes (20 displayed). Black traces show average waveforms from all spikes. Bottom-left: average waveforms superimposed. Right: auto-correlograms (top and centre) and ISI distribution (bottom) of the tracked unit under the two conditions. **C**, Summary data (mean ± SEM, 12 fix-free recording pairs) showing the percentage of head-fixed single units with an accepted match in the freely moving recording (same probe). Control data were generated by applying the same matching algorithm to recordings from different animals (different probes, 10 pseudo-random pairs). **D,** Percentage distribution of head-fixed single units with an accepted match in the freely moving recording located either on the same channel (channel difference = 0) or separated by 1 to 4 channels. Maximum accepted distance was 2 recording channels on the Neuronexus (50 µm) and 4 on the Neuropixels (40 µm vertical) probe.

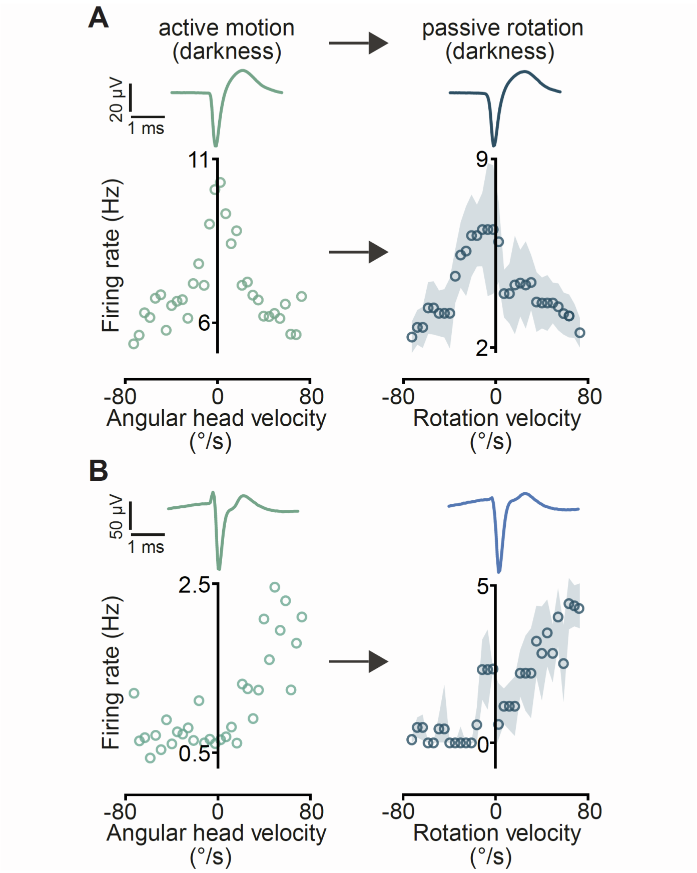

Figure S4. AHV cells maintain their tuning during restrained passive motion. Related to Figure 2.

A, Example of a bidirectional negatively correlated AHV cell, and **B**, a unidirectional AHV cell recorded during both open field exploration (left) and head-fixed passive rotation (right) in darkness. Top traces show average spike waveforms. Circles and shaded area on the right show trial-averaged firing rates (12 trials) and SEM, respectively.

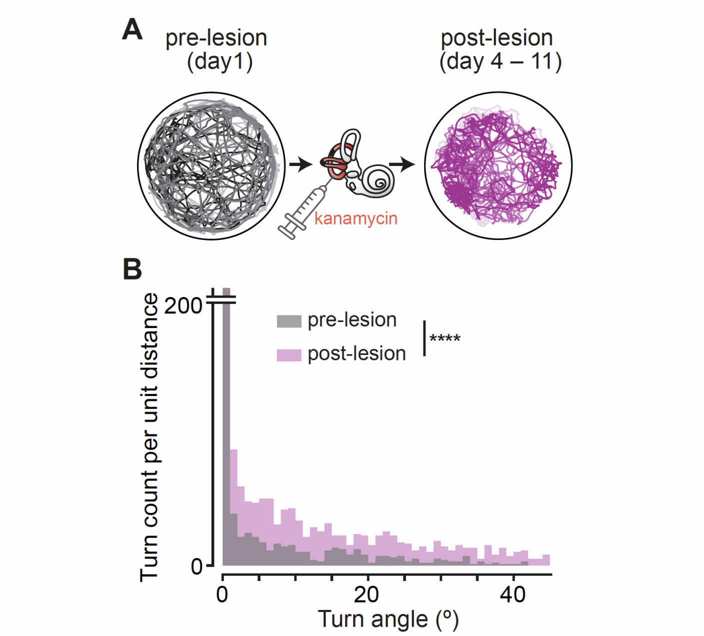

Figure S5. Locomotion and turning behaviour following vestibular lesions. Related to Figure 2.

A, Overlaid open field trajectories of 4 mice before (left) and after (right) bilateral kanamycin-induced lesions of the horizontal and posterior semi-circular canals. **B**, Scaled histogram of turning angles, binned at 1º, for all 4 mice before (grey) and after (pink) vestibular lesions. ****p = 2e-83, Kolmogorov–Smirnov test.

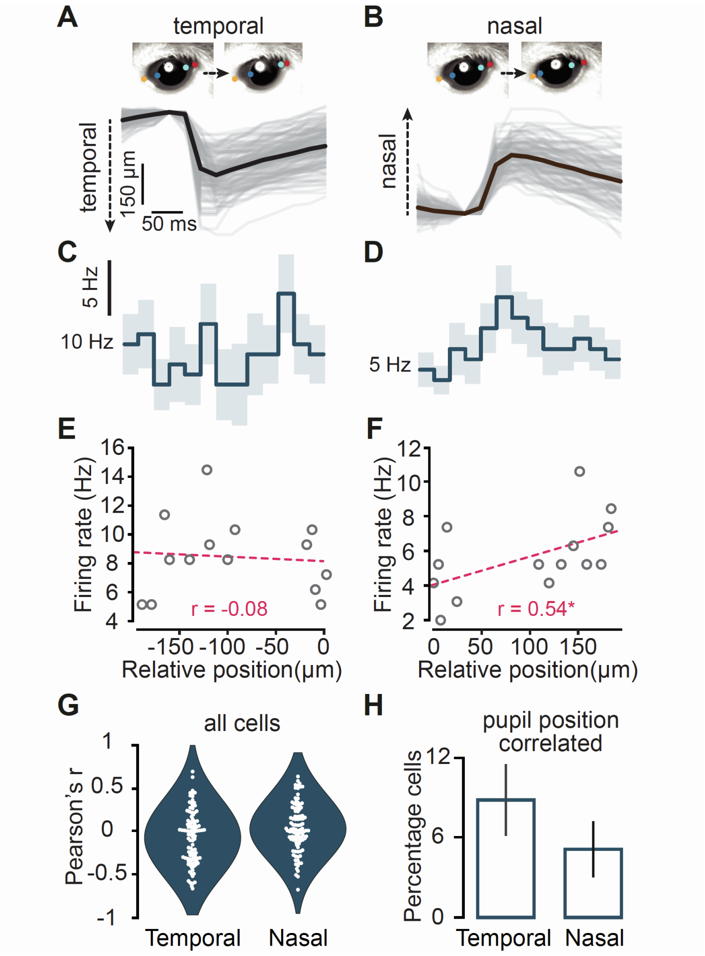

Figure S6. Eye movement-related activities in the RSP recorded during passive rotation. Related to Figure 2.

A-B, Top: video frames showing example temporal (**A**) and nasal (**B**) eye movements recorded at 40 fps in the dark. Coloured circles show marked positions for eye tracking using DLC. Bottom: example individual (grey), and averaged (black) fast eye movement events from one mouse. **C-D**, Mean (lines) and SEM (shades) firing rate histograms of an example cell aligned with detected temporal and nasal eye movements in **A** and **B**. Spiking rates during each eye movement event (from 75 ms before movement onset to 250 ms after) were determined in 25 ms time bins. **E-F,** Mean firing rate of the same cell (**C-D**) at each 25 ms time bin plotted against the averaged relative eye position. Dashed line shows linear fits. *Significant Pearson correlation ($p = 0.048$). **G**, Population data showing magnitude of correlations between firing rate and eye position. Circles represent individual neurons. **H**, Summary data (mean ± SEM) showing proportion of cells with significant eye-position correlations ($n = 3$ mice, 101 cells, p significance threshold = 0.05).

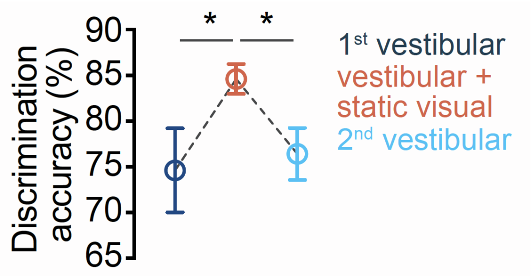

Figure S7. Angular velocity discrimination immediately before and after the addition of a visual cue. Related to Figure 4.

Mean (± SEM) discrimination accuracies for the 30:10 stimulus pair (average of 5 blocks) in all 5 mice tested first in the dark (1st vestibular), then under the multisensory condition (vestibular + static visual), and again under another "vestibular" condition (2nd vestibular). *p (1st vestibular vs. vestibular + visual) = 0.03, *p (2nd vestibular vs. vestibular + visual) = 0.04, one-way ANOVA with Holm-Sidak's test.

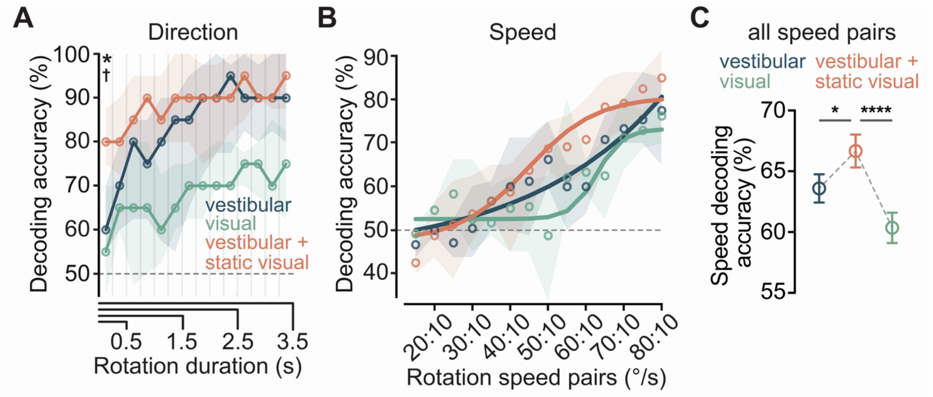

Figure S8. Combination of vestibular and visual stimuli improves decoding of angular self-motion by RSP neuronal populations. Related to Figure 6.

A, Population decoding accuracy for direction as a function of rotation duration. Circles and shaded areas show median and IQRs. Dashed line represents theoretical chance. *p (vestibular v. vestibular + visual) = 0.04, †p (visual v. vestibular + visual) = 0.01, pairwise Wilcoxon signed rank test with Bonferonni correction, first 250 ms, n = 10 mice, 19 recordings, 10 – 84 cells per recording. **B**, Population decoding accuracies for speed. Circles and shaded areas show median and IQRs. Lines are sigmoid fits. n = 7 mice, 12 recordings, 22 – 84 cells per recording. **C**, Mean (± SEM) speed decoding accuracy of all speed pairs. *p = 0.01, ****p = 6.4e-5, one-way ANOVA with Holm-Sidak's test.
